# Evaluation of desmoglein 1 and 3 autoantibodies in pemphigus vulgaris: correlation with disease severity

**DOI:** 10.4317/jced.56289

**Published:** 2020-05-01

**Authors:** Zahra Delavarian, Pouran Layegh, Atessa Pakfetrat, Nazila Zarghi, Mahboubeh Khorashadizadeh, Ala Ghazi

**Affiliations:** 1 Oral and Maxillofacial Diseases Research Center, Mashhad University of Medical Sciences, Mashhad, Iran; 2 Cutaneous Leishmaniasis Research Center, Mashhad University of Medical Sciences, Mashhad, Iran; 3 Faculty member of Mashhad University of Medical Sciences, Education Development Center (EDC), Mashhad, Iran; 4 Dentist, Mashhad, Iran

## Abstract

**Background:**

Pemphigus is an autoimmune blistering disease of the skin and mucous membranes caused by autoantibodies against desmoglein 1 (Dsg1) and desmoglein 3 (Dsg3). Pemphigus vulgaris (PV) is the most common form of pemphigus. The aim of this study was to assess the correlation between the levels of anti-desmoglein 1 and 3 autoantibodies and the severity of PV disease.

**Material and Methods:**

Nineteen newly diagnosed patients with pemphigus vulgaris were enrolled in this study. The titers of Dsg in subjects by using enzyme-linked immunosorbent assay (ELISA) were done at diagnosis time-point, 4th and 8th weeks after the initiation of treatment, and the correlation of antibodies with the oral and skin disease severity was evaluated.

**Results:**

The severity of cutaneous lesions was significantly correlated with anti-Dsg1 titer in all visits and the severity of mucosal lesions was correlated with the titer of Dsg3 in the third visit (<0.001, 0.001, 0.016 and 0.015 *P* value, respectively).

**Conclusions:**

Anti-Dsg-1 autoantibodies titers seem to be more useful in showing the extent of the disease and activity in pemphigus with mucocutaneous lesions.

** Key words:**Pemphigus vulgaris, Desmoglein (Dsg), Enzyme-linked immunosorbent assay (ELISA).

## Introduction

Pemphigus is a group acquired autoimmune bullous skin disease characterized by the presence of IgG auto-antibodies against keratinocyte cell surfaces of intercellular junctions. This leads to the loss of normal epithelial cell-to-cell adhesion (termed acantholysis) (1,2). Pemphigus affects 0.1-5.5% of the population per 100,000 per year (3,4). The two main types of pemphigus are pemphigus vulgaris (PV) and pemphigus foliaceus. The other forms include erythematosus, vegetans, IgA pemphigus, drug-induced pemphigus and paraneoplastic pemphigus (5,6). PV is the most common form of pemphigus, accounting for more than 80% of cases (4). PV is commonly linked to auto-antibodies against desmoglein 3 and in some cases, desmoglein 1. In PV patients, blisters are developed just superior to the basal cell layer in the epidermis causing chronic painful erosions in the oral cavity and flaccid blisters on normal-appearing skin (1,2,7). Clinical and histological examination, direct and indirect immunofluorescence and enzyme-linked immunosorbent assays (ELISAs) are used in the diagnosis of pemphigus (8,9). Standard treatments for pemphigus are corticosteroids and immunosuppressive drugs. The patients’ responses to treatment vary per case and frequent clinical relapses are reported (1,10,11). Therefore, clinical follow-ups are advised and serum anti-Dsg antibody levels should be monitored. For follow-up and therapeutic management of pemphigus patients, autoantibody titers, in particular, have been suggested. Hence, in this study, in order to evaluate the effectiveness of the ELISA assay as a follow-up tool for the management of pemphigus therapy, we sought to determine the titer of anti-desmoglein 1 and 3 auto-antibodies at the onset of the disease and during follow-up period (4th and 8th weeks after the initiation of treatment) and assess its association with the severity of the disease.

## Material and Methods

This study was conducted on newly diagnosed patients with PV who referred to Qaem Hospital, Imam Reza Hospital and Oral Medicine Department of Mashhad Dental School. The study protocol was approved by the institutional ethical committee. Diagnosis of PV was performed based on clinical examination, histopathology, and direct immunofluorescence.

Patients were selected based on the following criteria: Personal consent for entering the study, clinical confirmation of PV based on histopathology and direct immunofluorescence, in addition to having no previous treatment of lesions before entering the study. The criteria for excluding patients from the study included lack of participation in all follow up sessions, other types of pemphigus (pemphigus foliaceus or erythematosus).

Based on the inclusion and exclusion criteria, 19 patients were enrolled in this study. Demographic information including age, sex, and PV phenotype (mucosal, and mucocutaneous) were fully recorded. The severity score for both mucosal and cutaneous involvement was calculated as well. For all patients, the procedure was described and a written consent was obtained from each patient. 5 cc blood samples were collected from the patients. Samples were stored at -70 ° C until the laboratory investigation were done. Because the sampling time is effective in response due to circadian variation, samples were collected between 09:00-11:00. Serum samples were collected before the treatment and after the first and the second follow-ups. Given that the average recovery time is usually between the third and the fifth weeks after the beginning of treatment, the first follow-up antibody titration from the patients was performed in the fourth week after the beginning of the treatment and the second titration in the eighth week.

To detect autoantibodies by ELISA, anti-desmoglein 1 and 3 recombinant proteins were used (Euroimmun, Lübeck, Germany). Following the manufacturer’s instructions, a cut-off value of>20 U/mL considered positive. In order to determine the correlation between the levels of anti-desmoglein 1 and 3 autoantibodies and the severity of mucosal and cutaneous involvement, the severity of pemphigus disease at the onset and during the follow ups was calculated as follows (12,13):

Oral mucosa score:

0= No lesion.

1 = Minor activity (only buccal mucosal, lingual, labiogingival, palatal or pharyngeal involvement).

2 = Moderate activity (buccal and labiogingival, lingual, palatal or pharyngeal involvement).

3 = Severe (extensive oral erosions with >3 mucosal sites involved).

Skin score:

0= No lesion.

1= Up to five blisters and/or erosions.

2= 5–20 blisters and/or erosions.

3= >20 or extensive and confluent blisters and/or erosions.

Meanwhile, all patients were treated by a dermatologist with systemic corticosteroid (1-2 mg / kg) and an adjuvant mainly azathioprine (2-3 mg/kg) or mycophenolate mofetil (2gr/day).

Descriptive and inferential statistics were used to analyze the data. Central dispersion indices, mean and standard deviation were used to describe the data. Data were analyzed using Chi-square, Spearman, Mann-Whitney and Friedman correlation coefficients. In all tests, a significant level of 0.05 was considered with a confidence level of 0.95.

## Results

This study was conducted on 19 patients diagnosed with PV, including 9 males and 10 females. The patients varied in age between 17-73 years and the mean age of patients was 47.26 ± 15.11.

Six patients (31.6%) had only mucosal lesions, and in 13 patients (68.4%) both skin and oral mucosa were involved. Baseline characteristics of the subjects are provided in Table 1.

**Table 1:**
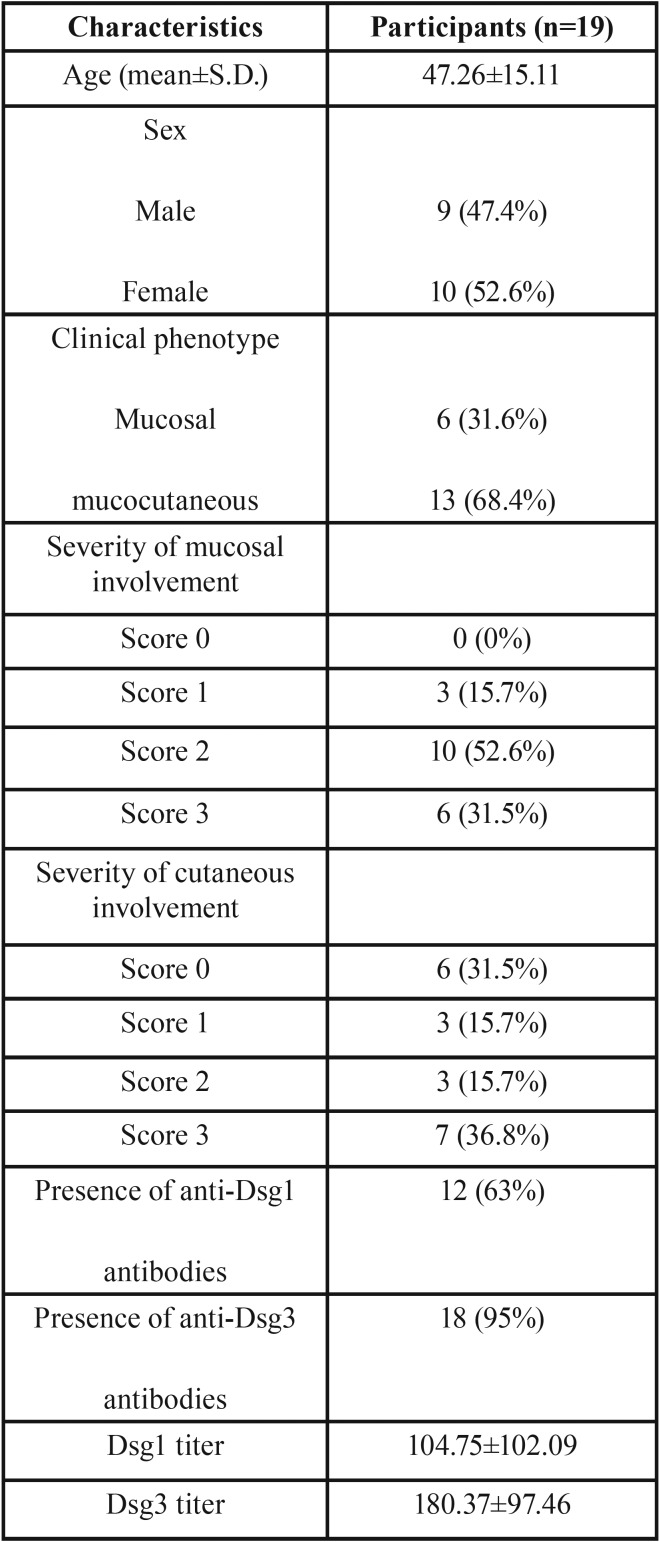
Baseline characteristics of the study participants.

Among 19 patients with PV, ELISA identified positive values of anti-Dsg3 auto-antibodies in 18 (95%) patients at the time of the initial diagnosis and in 16 (84%) and 11 (57%) after the first and the second follow ups, respectively. Positive values of anti-Dsg1 auto-antibodies were detected in 12 (63%) patients with PV at the time of diagnosis and in 8 (42%) and 11 (57%) patients after the first and the second follow ups, respectively (Table 2).

**Table 2:**
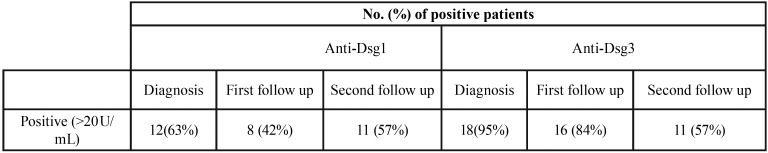
Presence of anti-Dsg3 and anti-Dsg1 antibodies in PV patients in the diagnosis and follow up sessions.

The average Dsg3 titer from the baseline (the diagnosis time-point) until the third session (second follow-up) was downregulated. The average titer of Dsg1 was downregulated from the first session (diagnosis) to the second session (second follow-up) and upregulated from the second to the third session ( Table 3).

**Table 3:**
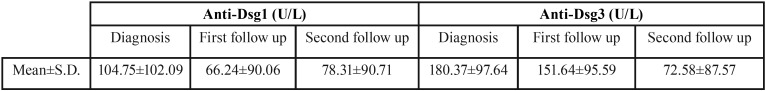
Mean of anti-Dsg3 and anti-Dsg1 antibodies in PV patients in the diagnosis and follow up sessions.

Based on the Wilcoxon test, there was a significant decrease for the mean titer of anti-Dsg1 auto-antibodies between the first and the second sessions (*P* = 0.006) and between the first and the third sessions (*P* = 0.025), but the change between the second and third sessions (*P* = 0.918) was not meaningful. The mean titer of anti-Dsg3 auto-antibodies showed a significant decrease among all sessions, such that between the first and the second sessions (*P* = 0.005), between the first and third sessions (*P* <0.001) and between the second and third sessions (*P* = 0.001), there was a significant difference.

**Figure 1:**
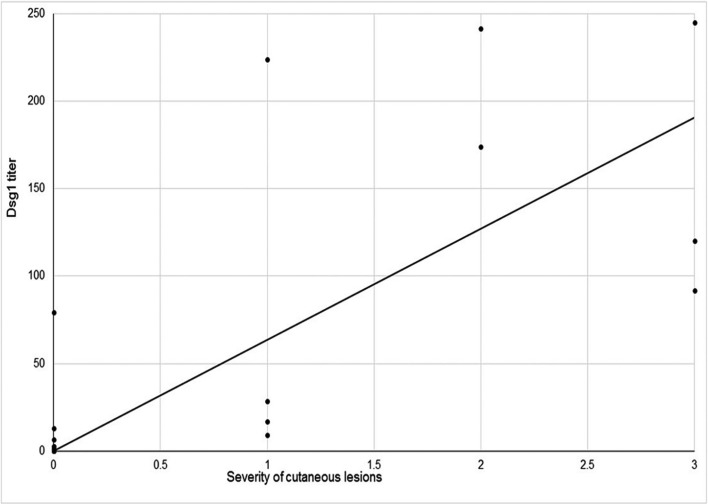
Association between severity of cutaneous lesions and Dsg1 titers in pemphigus vulgaris (*P* < 0.001, r = 0.51) in the first follow up session.

According to the Spearman test, in all sessions, the correlation between the Dsg1 titer and the severity of cutaneous lesions as well as between the titer of Dsg3 in the third session and the severity of mucosal lesions was statistically significant (*P* value <0.001, 0.001, 0.016 and 0.015, respectively). However, the titer of Dsg3 with the severity of cutaneous lesions, the titer of Dsg1 with the severity of mucosal lesions, and the titer of Dsg3 with the severity of mucosal lesions in the first and the second sessions did not show a significant relationship ( Table 4, Fig. 1).

**Table 4:**
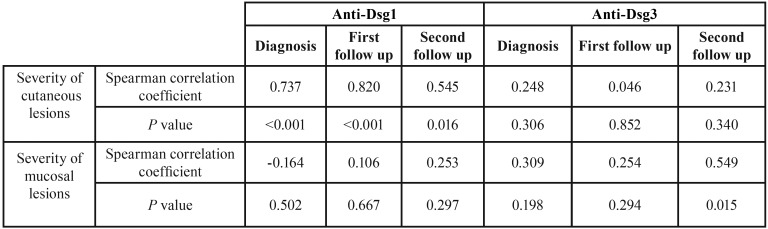
Relationship between anti-Dsg3 and anti-Dsg1 auto-antibodies and the severity of skin/ mucousal involvement during diagnosis and follow-up sessions based on the Spearman test.

## Discussion

Pemphigus is a potentially lethal mucocutaneous blistering disease whose early diagnosis and timely treatment is critical for the patients. The clinical course of pemphigus varies significantly, and it is a challenge to predict it only on the basis of clinical evaluation (1). Therefore, it can be helpful to apply a serial detection of anti-Dsg titers with the purpose of monitoring the disease activity in addition to managing the therapy.

The aim of this investigation was to evaluate the correlation between serum desmoglein 1 and 3 auto-antibodies and the severity of disease in patients with pemphigus vulgaris. In our study, 19 patients with PV were examined for anti-Dsg1 and anti-Dsg3 auto-antibodies titration by the ELISA method and their association with the severity of disease was assessed at the time-point of diagnosis, and after 4th and 8th weeks.

Overall, we found a significant correlation between the anti-Dsg1 auto-antibody titer and the severity of cutaneous lesions in all sessions as well as between the titer of anti-Dsg3 and the severity of mucosal lesions in the third session.

Numerous studies have explored the relationship between desmoglein level and the severity of pemphigus disease. A number of authors have found that the levels of antibodies against Dsg1 was positively correlated with an increase in the severity of cutaneous lesions and elevated levels of anti-Dsg3 antibodies was found to correlate with the severity of mucosal involvement (12-16). In contrast, Belloni-Fortina A *et al.* reported a relationship between the titer of anti-Dsg3 and the severity of both cutaneous and mucosal lesions, while they have found that anti-Dsg1 titer relates only to the severity of mucosal lesions (17).

Moreover, Mortazavi *et al.* and De D *et al.* showed that the degree of skin damages significantly enhanced upon an increase in the anti-Dsg1 concentrations, but they found no significant correlation between anti-Dsg3 antibodies and the severity of oral mucosal lesions (18,19).

Abasq *et al.* have reported that in PV patients, the anti- Dsg3 titer is not necessarily linked to the clinical course of mucosal lesions (20). Furthermore, Daneshpazhuh *et al.* previously reported that Dsg3 index values had a correlation with the severity of both cutaneous and mucosal diseases, while the anti-Dsg1 antibody levels only correlated with the severity of cutaneous lesions (21).

These discrepancies could be attributed to the low sample size in each of the reported studies in addition to genetic variations, and the varying criteria that are used to assess the disease severity (1,22).

In conclusion, anti-Dsg1 auto-antibody titers seem to be more useful for monitoring the extent of the disease and activity in pemphigus with mucocutaneous lesions. The anti-Dsg3 auto-antibody titers can be used for diagnostic purposes, but it fails to indicate disease activity. Multi-centre studies with a larger number of patients and a long-term follow-up may provide additional information for the interpretation of findings in association with the disease course.
